# Serum granulocyte-macrophage colony-stimulating factor (GM-CSF) is increased in patients with active radiographic axial spondyloarthritis and persists despite anti-TNF treatment

**DOI:** 10.1186/s13075-022-02888-6

**Published:** 2022-08-16

**Authors:** Charalampos Papagoras, Styliani Tsiami, Akrivi Chrysanthopoulou, Ioannis Mitroulis, Xenofon Baraliakos

**Affiliations:** 1 First Department of Internal Medicine & Laboratory of Molecular Hematology, University Hospital of Alexandroupolis, Democritus University of Thrace, Alexandroupolis, Greece; 2 Rheumazentrum Ruhrgebiet, Herne, Ruhr-University Bochum, Bochum, Germany; 3 Department of Biological Applications and Technology, University of Ioannina, Ioannina, Greece

**Keywords:** Radiographic axial spondyloarthritis, Granulocyte-monocyte colony-stimulating factor, Neutrophils

## Abstract

**Background:**

Accumulating evidence supports the role of monocytes and neutrophils in radiographic axSpA (r-axSpA). Granulocyte-macrophage colony-stimulating factor (GM-CSF) is a growth factor for both leukocyte lineages and a pro-inflammatory cytokine activating myeloid cells and promoting osteoclastogenesis. It acts through the JAK-STAT pathway. We measured serum GM-CSF and markers of bone metabolism in patients with r-axSpA before and after anti-TNF treatment.

**Methods:**

Patients with active r-axSpA despite treatment with NSAIDs, all eligible for treatment with a biologic agent, were recruited. Healthy donors were sampled as controls. Serum was collected before (baseline) and after 4–6 months (follow-up) of anti-TNF treatment and the following molecules were measured with ELISA: GM-CSF, sclerostin (SOST), and dickkopf-1 (Dkk-1).

**Results:**

Twelve r-axSpA patients (7 males, 5 females, median age 37 years) with a median disease duration of 1 year and 16 age- and sex-matched controls were included. At baseline, patients had mean BASDAI 6.3±2 and ASDAS 3.2±0.7, which decreased to 4.1±1.7 and 2.2±0.6 at follow-up, respectively. At baseline, r-axSpA patients had significantly higher mean serum levels of GM-CSF (150 vs 62pg/ml, *p*=0.049), significantly lower Dkk-1 (1228 vs 3052pg/ml, *p*=0.001), but similar levels of SOST (369 vs 544pg/ml, *p*=0.144) compared to controls. Anti-TNF treatment did not affect GM-CSF, Dkk-1, or SOST levels. Spearman correlation analysis showed that GM-CSF correlated positively with ASDAS at baseline (*r*=0.61, *p*=0.039), while no correlations were identified between bone markers (Dkk-1, SOST) on one hand and GM-CSF or disease activity indices on the other.

**Conclusions:**

GM-CSF is increased in patients with active AS and strongly correlates with disease activity. TNF inhibition does not affect GM-SCF levels, despite improving disease activity. GM-CSF may represent an important pathway responsible for residual inflammation during TNF blockade, but also a potential target of JAK inhibitors, explaining their efficacy in r-axSpA.

**Supplementary Information:**

The online version contains supplementary material available at 10.1186/s13075-022-02888-6.

## Background

Radiographic axial spondyloarthritis (r-axSpA), also known as ankylosing spondylitis, is the main representative of the axSpA family and a chronic inflammatory disease characterized by inflammation of spinal structures and peripheral entheses, which gradually results in aberrant bone formation creating syndesmophytes and enthesophytes [1]. In recent years, research has focused on various lymphoid cell populations as drivers of the inflammatory aspect of the disease, mainly through the interleukin (IL)-23/IL-17 axis [2].

Nevertheless, there is now increasing evidence that myeloid cells, such as neutrophils and macrophages, are also involved in both the inflammatory and osteoproliferative processes of axSpA. Disease activity in r-axSpA has been positively correlated with the proportion of neutrophils in the peripheral blood [3, 4], as well as with the serum levels of neutrophil extracellular traps (NETs), a product of inflammation-induced neutrophil death [5]. Moreover, neutrophils also appear to be the prevalent inflammatory cell type at the earliest stages of enthesitis [6], while in apophyseal joints of patients with r-axSpA, neutrophils are the cells with the highest IL-17 expression [7]. In this context, NETs carrying IL-17 were shown to promote mesenchymal stem cell differentiation towards osteoblasts in vitro [8]. On the other hand, cells of the monocytic lineage act either in a pro-inflammatory (M1 phenotype) or an anti-inflammatory/reparative fashion, while they can also differentiate to osteoclasts, thus participating in bone remodeling [9].

Granulocyte-macrophage colony-stimulating factor (GM-CSF) is a hematopoietic growth factor for both neutrophils and monocytes, acts as a pro-inflammatory cytokine inducing myeloid mobilization, activation, and cytokine production, and it is also involved in osteoclast differentiation [10].

In addition, molecules inhibiting the bone anabolic process, such as the Wnt pathway inhibitors sclerostin (SOST) and dickkopf-1 (Dkk-1) display altered expression and/or function in patients suffering from r-axSpA. Indeed, SOST expression is impaired in patients with r-axSpA, whereas low levels of sclerostin are linked to increased structural damage and progression [11]. Moreover, Dkk-1 has been shown to be dysfunctional in r-axSpA patients, although its serum levels show variable alterations [12, 13].

We therefore sought to conduct a pilot study to measure serum GM-CSF levels in patients with r-axSpA before and after treatment with a tumor necrosis factor (TNF) blocker and explore for correlations between GM-CSF levels, disease activity, and serum markers of inflammation and bone remodeling.

## Methods

The study included biologic-naïve patients diagnosed with r-axSpA with active disease despite treatment with non-steroidal anti-inflammatory drugs (NSAIDs), all being eligible for treatment with a biologic disease-modifying anti-rheumatic drug (bDMARD) and with the decision for treatment with a TNF inhibitor made by the treating rheumatologist independent of this analysis. Serum was collected before (baseline, BL) and after 4-6 months (follow-up, FU) of TNF inhibitor treatment. Age and sex-matched healthy donors were sampled as controls. The following molecules were measured in sera using ELISA: GM-CSF, SOST, and Dkk-1, according to the manufacturer’s instructions (R&D Systems). All subjects gave their written informed consent and the study was approved by the Institutional Boards of the Ruhr-University Bochum, Germany, and University Hospital of Alexandroupolis, Greece. Statistical analysis was performed using Fisher’s exact test, Mann-Whitney *U* test, and Spearman’s rank order correlation test on the Statistica software. The level of significance was set at *p*<0.05.

## Results

### Baseline demographics and clinical response

Twelve patients with r-axSpA (7 males, 5 females) with a median age of 37 years (range 22–52) and 16 controls (11 males, 5 females) with a median age of 37 years (range 22–55) were included. Median duration of r-axSpA symptoms was 6.5 years (range 0.5–29), while the median time since r-axSpA diagnosis was 1 year (range 0.5–25). Eight (66.7%) patients were HLA B27 positive. At BL, r-axSpA patients had a mean Bath Ankylosing Spondylitis Disease Activity Index (BASDAI) 6.3±2 and Ankylosing Spondylitis Disease Activity Score (ASDAS) 3.2±0.7. At FU, the mean BASDAI decreased to 4.1±1.7 and ASDAS decreased to 2.2±0.6 (Table 1).

**Table 1:** Characteristics of patients and controls and markers of r-axSpA disease activity before and after anti-TNF treatment

	Patients		Controls	*p*
*N*	12		16	
Males (*N*)	7		11	0.7
Median age, years (range)	37 (22–52)		37 (22–55)	0.65
Median disease duration since diagnosis, years (range)	1 (0.5–25)		NA	
Median disease duration since symptom onset, years (range)	6.5 (0.5–29)		NA	
HLA B27 positive, *N* (%)	8 (66.7)		ND	
	*Baseline*	*Follow-up*		
Mean CRP (SD), mg/dL	0.58 (0.5)	0.16 (0.2)	ND	
Mean BASDAI (SD)	6.3 (2)	4.1 (1.7)	NA	
Mean ASDAS (SD)	3.2 (0.7)	2.2 (0.6)	NA	

*ASDAS*, Ankylosing Spondylitis Disease Activity Score; *BASDAI*, Bath Ankylosing Spondylitis Disease Activity Index; *CRP*, C-reactive protein; *r-axSpA*, radiographic axial spondyloarthritis; *NA*, not applicable, *ND*, not done; TNF, tumor necrosis factor

### Levels of biomarkers and correlation with disease activity under treatment with TNF-blockers

**Fig. 1:**
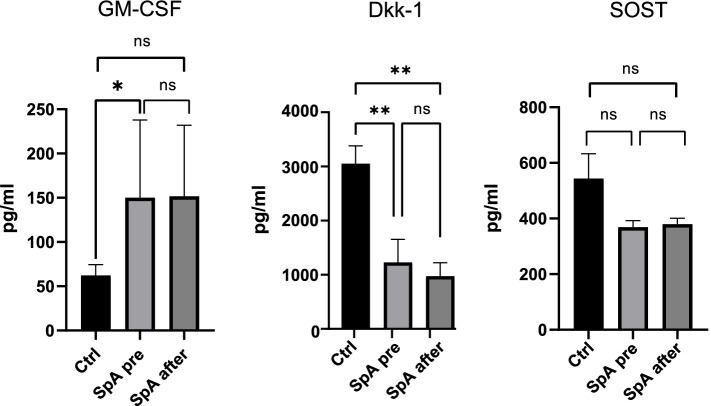
Serum levels of granulocyte-macrophage colony-stimulating factor (GM-CSF), sclerostin (SOST), and dickkopf-1 (Dkk-1) in patients with r-axSpA (*N*=12) before and after anti-TNF treatment compared to age and sex-matched healthy controls (*N*=16). *p<0.05 and **p≤0.001. Mann-Whitney test

At BL, serum levels of GM-CSF were significantly higher in r-axSpA patients (150 vs 62pg/ml, *p*=0.049), the levels of Dkk-1 were significantly lower (1228 vs 3052pg/ml, *p*=0.001), whereas the levels of SOST were not different (369 vs 544pg/ml, *p*=0.144) compared to controls. At FU, the r-axSpA patient mean serum levels of GM-CSF were 152pg/ml, of Dkk-1 975pg/ml, and of SOST 379pg/ml, none of which showed significant change compared to BL (Fig. 1). Spearman correlation analysis among all variables both at BL and FU showed that GM-CSF levels were positively correlated with ASDAS at baseline (*r*=0.61, *p*=0.039), but not at FU (*r*=0.420, *p*=0.227). They were also negatively correlated with age (*r*=− 0.68, *p*=0.018), but not with disease duration since diagnosis (*r*=− 0.27, *p*=0.400) or symptom duration (*r*=− 0.004, *p*=0.991). Notably, a significant negative correlation between GM-CSF levels and age was found in healthy controls (*r*=− 0.700, *p*=0.003), as well. No correlations were identified between serum levels of Dkk-1 and SOST on one hand and GM-CSF levels, disease characteristics or activity indices on the other.

## Discussion

In this pilot study, we demonstrate that clinically active non-bDMARD-treated r-axSpA patients have significantly higher serum levels of GM-CSF than healthy controls. In addition, GM-CSF levels positively correlated with ASDAS in these patients prior to treatment initiation. Interestingly, although after 4–6 months of treatment with TNF blockers patients improved clinically (as expressed by BASDAI and ASDAS), GM-CSF levels remained unaffected, while the correlation with ASDAS was abolished. This finding suggests that GM-CSF is increased in r-axSpA and persists despite the suppression of the TNF-mediated inflammatory pathways. As a consequence, increased GM-CSF keeps on enhancing myelopoiesis and the pro-inflammatory functions of myeloid cells, including neutrophils and monocytes. This may explain the residual disease activity despite anti-TNF treatment, since also at the same time both mean BASDAI and ASDAS values decreased but not to a status of ‘low’ disease activity (BASDAI <4 or ASDAS <2.1). On the other hand, while GM-CSF levels did not correlate with r-axSpA symptom duration or time since r-axSpA diagnosis, they inversely correlated with age. However, the same correlation was also observed in healthy controls, suggesting that this observation is not r-axSpA-specific, but may be a normal finding.

Several studies reported a positive correlation between peripheral neutrophil-to-lymphocyte ratio and clinical disease activity [3, 4], which may reflect enhanced granulopoiesis, induced by pro-inflammatory mediators, including GM-CSF. In the same line, the higher levels of circulating NET remnants in active r-axSpA also suggest a state of increased inflammatory activity of neutrophils, which are prone to formation of NETs decorated with IL-1β and IL-17 [5, 8]. Concerning molecules regulating bone remodeling (Dkk-1 and SOST), we found lower levels of Dkk-1 in patients with r-axSpA, as compared to controls, which remained unaffected by anti-TNF treatment. This observation is in line with previous reports [13] and suggests that in r-axSpA the Wnt-dependent bone anabolic process is not effectively regulated, resulting in an increased bone formation [14]. Although no correlations between clinical indices, GM-CSF and Dkk-1 or SOST could be demonstrated, analysis of a wider array of biomarkers in samples from larger patient sets is needed to understand the mechanisms governing bone remodeling in r-axSpA.

The role of myeloid cells in the pathogenesis of spondyloarthritis (SpA) is gradually unveiled. A recent analysis of the transcriptome of peripheral monocytes from two axSpA cohorts revealed a skewing of monopoiesis towards granulocyte monocyte progenitors (GMPs) rather than monocyte dendritic cell progenitors, particularly in subjects with established r-axSpA versus non-radiographic axial SpA [15]. These observations could be attributed to increased GM-CSF activity. Indeed, the frequency of GMPs and neutrophils is increased in the bone marrow of the SKG SpA model, while GMP differentiation towards neutrophils is detected at the site of inflamed joints, suggesting that focal inflammation is associated with extra-medullary granulopoiesis. Importantly, treatment with an anti-GM-CSF antibody not only ameliorated the SpA phenotype in mice, but also decreased the proportion of GMPs in the bone marrow and joints, as well as their neutrophil output at both sites [16].

To date, several anti-GM-CSF monoclonal antibodies are being developed and some of them have successfully been tried in rheumatoid arthritis [10]. Moreover, suppression of the intracellular GM-CSF signaling by JAK inhibitors (which have no direct effect on TNF and IL-17 signaling), may also provide an explanation of the efficacy of those drugs for the treatment of r-axSpA [17].

The results presented above derive from a small and preliminary study with a short observation period. Consequently, since both GM-CSF measurements occurred only a few months apart, it is uncertain whether GM-CSF levels remain relatively stable or vary over years possibly with relation to disease activity or in response to different treatments. However, our results suggest a potential pathogenetic role of GM-CSF in r-axSpA and corroborate previous preclinical and clinical observations that support further studies on the biology and clinical utility of GM-CSF in this disease.

## Supplementary Information

**Additional file 1: Suppl. Table 1.** Single patient data.

## Data Availability

All data generated and analyzed during the study are included in this published article and its supplementary information files.

## Abbreviations

ASDAS: Ankylosing Spondylitis Disease Activity Score
BASDAI: Bath Ankylosing Spondylitis Disease Activity Index
bDMARD: Biologic disease-modifying anti-rheumatic drug
BL: Baseline
Dkk-1: Dickkopf-1
FU: Follow-up
GM-CSF: Granulocyte-Monocyte Colony Stimulating Factor
GMPs: Granulocyte monocyte progenitors
IL: Interleukin
NETs: Neutrophil extracellular traps
NSAIDs: Non-steroidal anti-inflammatory drugs
r-axSpA: Radiographic axial spondyloarthritis
SOST: Sclerostin
TNF: Tumor necrosis factor
