# Supplementary material for: Serum granulocyte-macrophage colony-stimulating factor (GM-CSF) is increased in patients with active radiographic axial spondyloarthritis and persists despite anti-TNF treatment
Source: Arthritis Res Ther. 2022 Aug 16;24:195. doi: 10.1186/s13075-022-02888-6 (PMC9380324; doi:10.1186/s13075-022-02888-6)
Supplement: Supplementary file 1 — Additional file 1: Suppl. Table 1. Single patient data. [file 13075_2022_2888_MOESM1_ESM.pdf]

| | | | Suppl. Table 1: Single patient data | | | | | | | | | | | | | | |
|---|---|---|---|---|---|---|---|---|---|---|---|---|---|---|---|---|---|
| **Patient ID** | **Sex** | **Age** | **Symptom Duration** | **Disease duration since diagnosis** | **GM-CSF (pg/ml)** | | **SOST (pg/ml)** | | **Dkk-1 (pg/ml)** | | **BASDAI** | | **ASDAS** | | **CRP** | |
| | | | | | **BL** | **FU** | **BL** | **FU** | **BL** | **FU** | **BL** | **FU** | **BL** | **FU** | **BL** | **FU** |
| 1. | M | 52 | 10.0 | 6.0 | 49 | 34 | 455 | 375 | 520 | 940 | 5.6 | 3.6 | 3.2 | 1.7 | 0.5 | 0.2 |
| 2. | F | 35 | 5.0 | 0.5 | 39 | 107 | 316 | 451 | 770 | 290 | 6.0 | | 3.0 | | 0.3 | |
| 3. | M | 39 | 7.0 | 1.5 | 69 | 50 | 324 | 396 | 190 | 520 | 7.5 | 7.4 | 3.6 | 3.2 | 0.3 | 0.5 |
| 4. | F | 52 | 29.0 | 25.0 | 58 | 37 | 280 | 367 | 5480 | 3290 | 10.8 | 3.7 | 3.4 | 1.7 | 0.5 | 0.1 |
| 5. | M | 23 | 11.0 | 0.5 | 72 | 70 | 436 | 276 | 2330 | 1380 | 3.0 | 3.8 | 1.7 | 1.9 | 0.1 | 0.0 |
| 6. | M | 22 | 4.0 | 0.5 | 1114 | 1008 | 476 | 535 | 1360 | 1110 | 7.9 | | 4.4 | | 1.2 | |
| 7. | F | 25 | 6.0 | 0.5 | 58 | 46 | 342 | 327 | 160 | 130 | 6.4 | 5.1 | 3.2 | 2.7 | 0.1 | 0.3 |
| 8. | F | 48 | 3.5 | 1.5 | 47 | 46 | 371 | 360 | 170 | 0 | 3.8 | 2.1 | 2.5 | 1.7 | 1.2 | 0.0 |
| 9. | F | 52 | 18.0 | 13.0 | 53 | 42 | 393 | 418 | 730 | 1000 | 6.5 | 5.9 | 3.1 | 2.6 | 0.0 | 0.1 |
| 10. | M | 40 | 0.5 | 0.5 | 53 | 56 | 251 | 251 | 680 | 1050 | 6.0 | 2.2 | 3.2 | 1.6 | 1.1 | 0.4 |
| 11. | M | 31 | 7.0 | 5.0 | 70 | 41 | 484 | 396 | 1500 | 1340 | 5.2 | 2.4 | 3.8 | 1.6 | 1.5 | 0.0 |
| 12. | M | 26 | 1.0 | 0.5 | 118 | 283 | 302 | 400 | 840 | 650 | 6.4 | 5.0 | 3.5 | 3.0 | 0.1 | 0.0 |

| Suppl. Table 2: Individual control data | | | | | |
|---|---|---|---|---|---|
| **Control ID** | **Sex** | **Age** | **GM-CSF (pg/mL)** | **SOST (pg/mL)** | **Dkk-1 (pg/mL)** |
| 1. | M | 25 | 67 | 513 | 3120 |
| 2. | M | 23 | 162 | 400 | 2550 |
| 3. | M | 52 | 27 | 789 | 1700 |
| 4. | F | 53 | 43 | 345 | 3200 |
| 5. | M | 42 | 27 | 305 | 4780 |
| 6. | F | 24 | 123 | 1505 | 2150 |
| 7. | F | 35 | 37 | 400 | 2950 |
| 8. | F | 45 | 43 | 415 | 4480 |
| 9. | M | 26 | 38 | 480 | 5520 |
| 10. | M | 55 | 31 | 502 | 4250 |
| 11. | F | 54 | 23 | 273 | 2420 |
| 12. | M | 32 | 46 | 469 | 1130 |
| 13. | M | 22 | 82 | 411 | 3180 |
| 14. | M | 53 | 30 | 287 | 3980 |
| 15. | M | 39 | 178 | 1298 | 2660 |
| 16. | M | 32 | 37 | 305 | 760 |
